# Primordial follicle activation in the ovary of Ames dwarf mice

**DOI:** 10.1186/s13048-014-0120-4

**Published:** 2014-12-29

**Authors:** Augusto Schneider, Xu Zhi, Fabiana Moreira, Thomaz Lucia, Rafael Gianella Mondadori, Michal M Masternak

**Affiliations:** Faculdade de Nutrição, Universidade Federal de Pelotas, Rua Gomes Carneiro, 1 Sala 239, CEP 96020-220 Pelotas, RS Brazil; College of Medicine, Burnett School of Biomedical Sciences, University of Central Florida, 6900 Lake Nona Blvd., Orlando, FL 32827 USA; Center of Reproductive Medicine, Department of Obstetrics and Gynecology, Peking University Third Hospital, Beijing, 100191 China; Faculdade de Veterinária, Universidade Federal de Pelotas, Pelotas, RS Brazil; Instituto de Biologia, Universidade Federal de Pelotas, Pelotas, RS Brazil; Department of Head and Neck Surgery, The Greater Poland Cancer Centre, 15 Garbary St., 61-866 Poznan, Poland

**Keywords:** Ames dwarf, Growth hormone, Ovaries, FOXO3a

## Abstract

**Background:**

The insulin receptor substrate 1 (IRS1), phosphoinositide 3-kinase (Pi3k), protein kinase B (Akt1), Forkhead Box O3a (FOXO3a) pathway is directly involved in aging and ovarian activation of follicle growth. Therefore, the aim of this work was to measure the expression of genes related to the ovarian pathway for activation of primordial follicles and FOXO3a protein phosphorylation between young and old female Ames dwarf (df/df) and normal (N) mice.

**Methods:**

For this study ovaries from N (n = 10) and df/df (n = 10) female mice were collected at 5–6 months of age and at 21–22 months of age. For immunohistochemistry ovaries from 12 month-old and df/df mice were used.

**Results:**

The expression of *Irs1*, *Pi3k*, *Akt1*, mammalian target of rapamycin (*Mtor*), suppressor of cytokine signaling −2 (*Socs2*), *Socs3* was lower (P < 0.05) in older than younger N mice and not different (P > 0.05) between young and old df/df mice. The expression of *Foxo3a* was also lower (P < 0.05) in old than younger N and df/df mice and was higher (P < 0.05) in old df/df than N mice. Expression of *Amh* was lower (P < 0.05) in old than young N and df/df mice and was higher (P = 0.0009) in df/df than N mice. Imunnostaining for p-FOXO3 was lower in df/df than N mice (P < 0.001), although FOXO3 immunostaining was not different (P > 0.05) between df/df and N mice.

**Conclusions:**

In sum, the present study indicates that lower expression of *Irs1*, *Socs2*, *Socs3*, *Akt1*, *Pi3k*, *Mtor* and *Foxo3a* mRNA in the ovaries of older mice of both genotypes is associated to a reduced ovarian activity revealed by lower expression of *Amh* mRNA. At the same time, ovaries of old df/df mice maintained higher expression of *Foxo3a* mRNA, which was associated to higher ovarian activity. We have shown that df/df females have a lower level of p-FOXO3 in oocytes from primordial/primary follicles, an important activator of follicular growth. Therefore, this study strongly indicates that Prop1^df^ mutation causes delayed ovarian aging.

## Background

The Ames dwarf mice (df/df) carry a mutation at the Prop1 (Prophet of Pit1) locus that impairs the development of the anterior pituitary gland [1], resulting in deficiency of growth hormone (GH), thyroid-stimulating hormone (TSH), and prolactin [2]. As the result of the GH deficiency these mice are characterized by severely low circulating insulin-like growth factor I (IGF-I) and reduced adult body size [3]. Importantly, regardless of these hormonal deficiencies, df/df mice live 35-75% longer than their normal littermates [4]. Similarly to the df/df mice, the knockout mice with a disruption of the GH receptor gene/GH binding protein (GHRKO) and normal mice (N) subjected to mild calorie restriction have reduced serum IGF-I concentrations and extended lifespan [5,6]. At the same time transgenic mice over-expressing GH are characterized by significant reduction of their lifespan when comparing to their normal controls [7]. Subjecting GH-deficient df/df mice to 6 weeks of GH replacement therapy early in life decreased the longevity of these long-living mutants to the lifespan maintained by N mice [8]. All these data indicate that the GH/IGF-I axis and its intracellular mediators play a major role in the process of aging and longevity [9]. However, regardless of severe reduction of IGF-I in circulation it was shown that there is compensatory, GH independent production of IGF-I in the hippocampus and ovaries [10,11].

It is well established in reproductive studies that a functional GH/IGF-I axis is important for normal ovarian function [12]. Although df/df female mice are infertile, they have normal estrous cycles and are able to ovulate [2]. The infertility probably arises from the inability to maintain the gestation, since normal pregnancy can be achieved after prolactin treatment, with an average 2.6 live offspring/pregnancy [13]. Pituitary production and circulating concentrations of follicle stimulating hormone (FSH) and luteinizing hormone (LH) are present but reduced in df/df mice [14]. GHRKO mice and N mice subjected to calorie restriction share several physiological characteristics with df/df mice; however, they are able to reproduce naturally, despite a reduced litter size, mainly due to a reduction in the number of large follicles and the ovulation rate [15,16]. One noteworthy point is the prolonged reproductive lifespan of GHRKO mice, as indicated by the presence of ovarian activity at a later age, when normal mice have already depleted the ovarian follicular reserves [17]. This condition seems to occur due to the reduction in the progression of follicles from the primordial to the primary stage in both GHRKO and calorie restriction mice [16,18], although no reports for df/df mice were found in the literature.

The ovarian primordial follicle reserve is established during fetal development [19]. After birth there is a continuous activation of the primordial follicle pool which leads to growth of individual follicles, while the rest of the pool remain quiescent for months or even years, until the reserve is depleted in a event known as menopause. Several local factors are believed to be involved in this activation process [19]. One of them is the activation of the phosphoinositide 3-kinase (Pi3k)/protein kinase B (Akt1) signaling pathway, an important regulator of cell proliferation and survival, that has been implicated in the activation of primordial follicles [20,21]. It is known that activation of the Pi3k/Akt1 pathway and the downstream transcription factor Forkhead Box O3a (FOXO3a) induced by the oocyte is a key regulator of the activation of primordial follicles [22]. The hyperactivation of Akt1 results in hyperphosphorylation and nuclear export of FOXO3a, culminating with the global activation of primordial follicles and premature ovarian failure [23]. The mammalian target of rapamycin (mTOR) pathway also has a direct relationship with activation of the Pi3k/Akt1 pathway [24]. mTOR activation accelerates primordial follicle activation in a synergistic way with the Pi3k/Akt1 pathway [25]. The IRS-Pi3k-Akt1-FOXO pathway is stimulated by both insulin and IGF-I and its reduced activation seems to be involved in the extended longevity observed in the mouse models mentioned before [26]. Therefore, it is possible that the extended reproductive lifespan in these mice can be also related to the components of this pathway.

Based on these evidences the aim of this work was to measure the expression of genes related to the ovarian pathway for activation of primordial follicles between young (6 months old) and old (22 months old) female df/df and N mice and also measure the level of FOXO3 and p-FOXO3 protein in the ovaries of 12-month old N and Ames dwarf mice.

## Methods

Normal (N; n = 10) and Ames dwarf mice (df/df; n = 10) (all females) were bred and maintained under temperature- and light-controlled conditions (22 ± 2°C, 12 hour light/12 hour dark cycle) [27]. The animals were anesthetized and euthanized after overnight fasting and the pair of ovaries was collected and stored at −80°C. The ovarian collection was performed at 5–6 months of age (young; 5 N and 5 df/df mice) and at 21–22 months of age (old; 5 N and 5 df/df mice).

The pair of ovaries was removed from the −80°C and homogenized with 400 μL of Qiazol (Qiagen, Valencia, CA, USA) using 0.5 mm zirconium oxide beads in the Bullet Blender 24 (Next Advance, Averill Park, NY, USA) for 4 minutes at speed 10. After that, 300 μL of Qiazol were added to the samples and incubated for 5 min at room temperature. Next 170 μL of chloroform was added, the samples were homogenized and incubated for 3 min at room temperature. Samples were then centrifuged at 4°C at 12000 *g* for 15 min and the clear upper phase transferred to a new tube with 525 μL of cold ethanol. The solution was transferred for a commercial column purification system (miRNeasy Mini Kit, Qiagen) and on-column DNase treatment (RNase-free DNase Set, Qiagen) following manufacturer's instructions was performed. The quantity of RNA was determined using a spectrophotometer (Epoch Microplate Spectrophotometer, Biotek, Winooski, VT, USA) and diluted to 200 ng/μL. Reverse transcription reactions were performed with 1 μg of RNA (5 μL) using iScript Synthesis Kit (Biorad, Hercules, CA, USA) in a 20 μL volume incubated at 5 min at 25°C, 30 min at 42°C and 5 min at 85°C (MJ Mini Personal Thermal Cycler, Biorad). The final cDNA solution was diluted to 10 ng/μL before use.

Real-time PCR using SYBR Green dye was used to evaluate gene expression. β_2_ microglobulin expression was used as an internal control. The primer sequences are listed in Table 1. The PCR reactions were performed in duplicate in a 20 μL volume using 5 μL of Fast SYBR Green Mastermix (Applied Biosystems, Foster City, CA, USA), 0.4 μL of each primer (10 μM stock) and 2 μL of cDNA. Fluorescence was quantified with the ABI Prism 7500 Fast Real Time PCR System (Applied Biosystems). For each assay 45 PCR cycles were run (95°C for 3 sec and 60°C for 30 sec) and a dissociation curve was included at the end of the reaction to verify the amplification of a single PCR product. Analyses of amplification plots were performed with the 7500 Software (Applied Biosystems). Each assay plate included a negative control. The coefficient of variation was below 5% for all the primer pairs used. Relative expression was calculated from the equation 2^A−B^/2^C−D^ (where A is the cycle threshold [Ct] number for the gene of interest in the first control sample; B, the Ct number for the gene of interest in the analyzed sample; C, the Ct number for β2 microglobulin in the first control sample; D, the Ct number for β2 microglobulin in the analyzed sample). The first control sample was expressed as 1.00 by this equation, and all other samples were calculated in relation to this value. Afterward, the results in the control group (normal young mice) were averaged, and all other outputs were divided by the mean value of the relative expression in the control group to yield the fold change of the genes of interest expression compared to the control group [28].

**Table 1:** **Primer pairs used in the experiment**

**Gene**	**Primer sequence**
*β2M*	*For* AAGTAT ACT CAC GCC ACC CA
	*Rev* CAG GCGTAT GTATCA GTC TC
GHR	*For* AGGTCTCAGGTATGGATCTTTGTCA
	GCCAAGAGTAGCTGGTGTAGCCT
IGF-I	*For* CTGAGCTGGTGGATGCTCTT
	*Rev* CACTCATCCACAATGCCT
SOCS2	*For* CTG CGC GAG CTC AGT CAA A
	*Rev* ATC CGC AGGTTA GTC GGT CC
SOCS3	*For* TGT CGG AAG ACT GTC AAC GG
	*Rev* GAA GAA GCC AAT CTG CCC CT
Akt1	*For* CCG GTT CTT TGC CAA CAT CG
	*Rev* ACA CACTCC ATG CTG TCA TCT T
Pi3k	*For* TAGCTGCATTGGAGCTCCTT
	*Rev* TACGAACTGTGGGAGCAGAT
mTOR	*For* CGG CAA CTT GAC CAT CCT CT
	*Rev* TGCTGG AAG GCGTCA ATC TT
IRS-1	*For* CGG GGA AGA CGA GAT GCT TT
	*Rev* TAC TGG AGC CTT GCG GCA C
FOXO3a	*For* TCCCAGATCTACGAGTGGATGG
	*Rev* CCTTCATTCTGAACGCGCAT
BMP15	*For* GAGCGAAAATGGTGAGGCTG
	*Rev* GGCGAAGAACACTCCGTCC
GDF9	*For* GCTCTATAAGACGTATGCTACC
	*Rev* CAGAGTGTATAGCAAGACCGAT
AMH	*For* TCCTACATCTGGCTGAAGTGATATG
	*Rev* CAGGTGGAGGCTCTTGGAACT

For the immunohistochemistry (IHC) analysis, ovaries from 12-month old N (n = 4) and Ames dwarf (n = 4) were collected and embedded in paraffin. After that, 3 μm slices obtained in an automatic microtome were adhered in slides impregnated by 3% organosilane (Sigma Chemical Company®, St. Louis, MO, USA) in ethanol. The samples were deparaffinized with xylene and rehydrated with graded alcohols. The primary polyclonal antibodies were: anti-Foxo3 phosphorylated (p-Foxo3) [p-FKHRL1 antibody; Ser 253, sc-101683-rabbit (IgG)] and anti-Foxo3 [FKHRL1 antibody; N16, sc-101683-goat (IgG)], both diluted 1:50. The primary antibodies were obtained from Santa Cruz Biotechnology (Santa Cruz, CA, USA) and diluted in 1.5% BSA solution. Blockage of the endogenous peroxidase activity was conducted with hydrogen peroxide block solution (Spring Bioscience, Pleasanton, CA, USA), whereas the antigenic recovery was performed in humid heat, during 3 min after the boiling point, in citrate solution pH 6.0. Non-specific background staining was reduced by covering the tissue sections that received protein block (Spring Bioscience, Pleasanton, CA, USA). Thereafter, slides were incubated overnight in a humid chamber at 4°C. Slides with p-Foxo3 antibody were instilled with Reveal Polyvalente HRP® Kit (Spring Bioscience, Pleasanton, CA, USA) and slides with anti-Foxo3 were instilled with the Histofine® kit (Nichirei Bioscience, Tokyo, Japan). Slides were incubated at room temperature, with 3,3’ diaminobenzidine (DAB-K3468, Dako Corporation, Carpinteria, CA, USA), counterstained with Mayer’s hemalum solution (Merck, Darmstadt, Germany) and mounted with coverslips and synthetic resin (Sigma Chemical Company®, St. Louis, MO, USA). The same technician conducted all procedures for IHC.

Images of ovarian sections were captured with a digital camera (Olympus DP72) attached to a compound light microscope (Olympus BX 51, Tokyo, Japan), using the 40 X objective. Only oocytes included in primordial, primary and secondary follicles were marked for image analysis and the intensity of labeling was captured. The oocytes were classified as: included in primordial/primary follicles (OIPF), when surrounded by one layer of flat or cubic granulosa cells and included in secondary follicles (OISF), when surrounded by two or more layers of cubic granulosa cells [29]. For the Foxo3 antibody a total of 28 OIPF (14 N and 14 df/df) and 8 OISF (4 N and 4 df/df) were analyzed; and for the evaluation of p-Foxo3 immunostaining 30 OIPF (15 N and 15 df/df) and 9 OISF (4 N and 5 df/df) were used. Protein quantification was performed by software image analysis [30-33]. The most common value (the mode) of each area was registered by the 32-bit histogram application of the Image J® software, using a scale ranging from 0 (the greatest staining intensity) to 255 (no staining) that was converted to a percentual scale from 0% (no staining) to 100% (greatest staining).

The results are presented as mean ± standard error of the mean (SEM). All statistical analyses were performed using Graphpad Prism 5 (Graphpad Software Inc., La Jolla, CA, USA). One-way ANOVA was performed to estimate between groups difference, followed by Tukey’s multiple comparison test. Immunostaining intensity for FOXO3 and p-FOXO3 was compared between strains by t-test. A P value lower than 0.05 was considered statistically significant.

All animal procedures employed in our presented work were approved by the Laboratory Animal Care and Use Committee (LACUC) at the Southern Illinois University School of Medicine (Springfield, IL).

## Results

**Figure 1:**
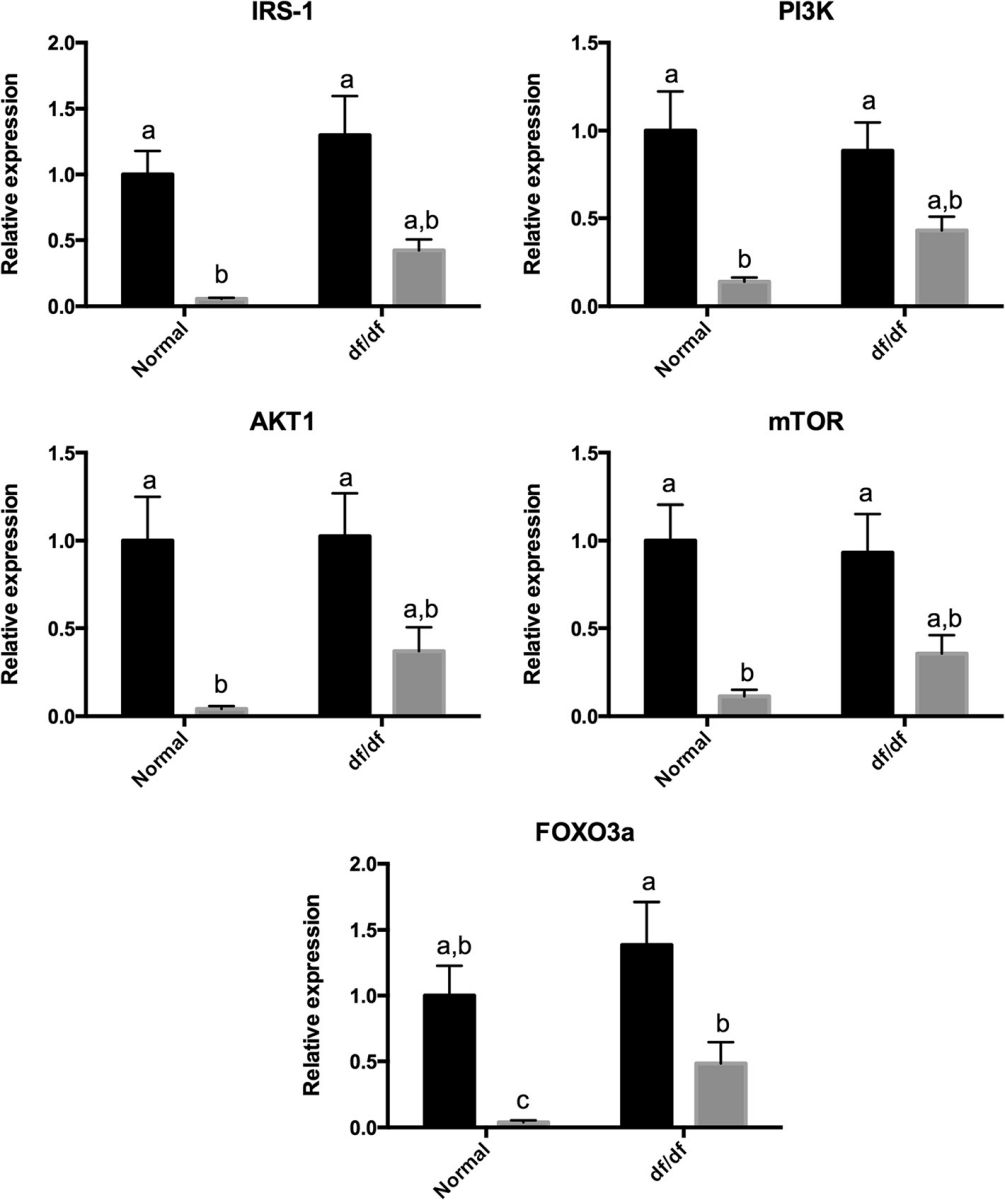
**Ovarian gene expression for genes in the insulin signaling pathway (IRS1, Pi3k, Akt1 and FOXO3a) in young (6 months–black bar) and old (22 months–grey bar) normal (N) and Ames dwarf (df/df) mice.** Different letter indicate significant differences (P<0.05). Expression of all genes showed was lower in old than younger mice (P<0.05).

**Figure 2:**
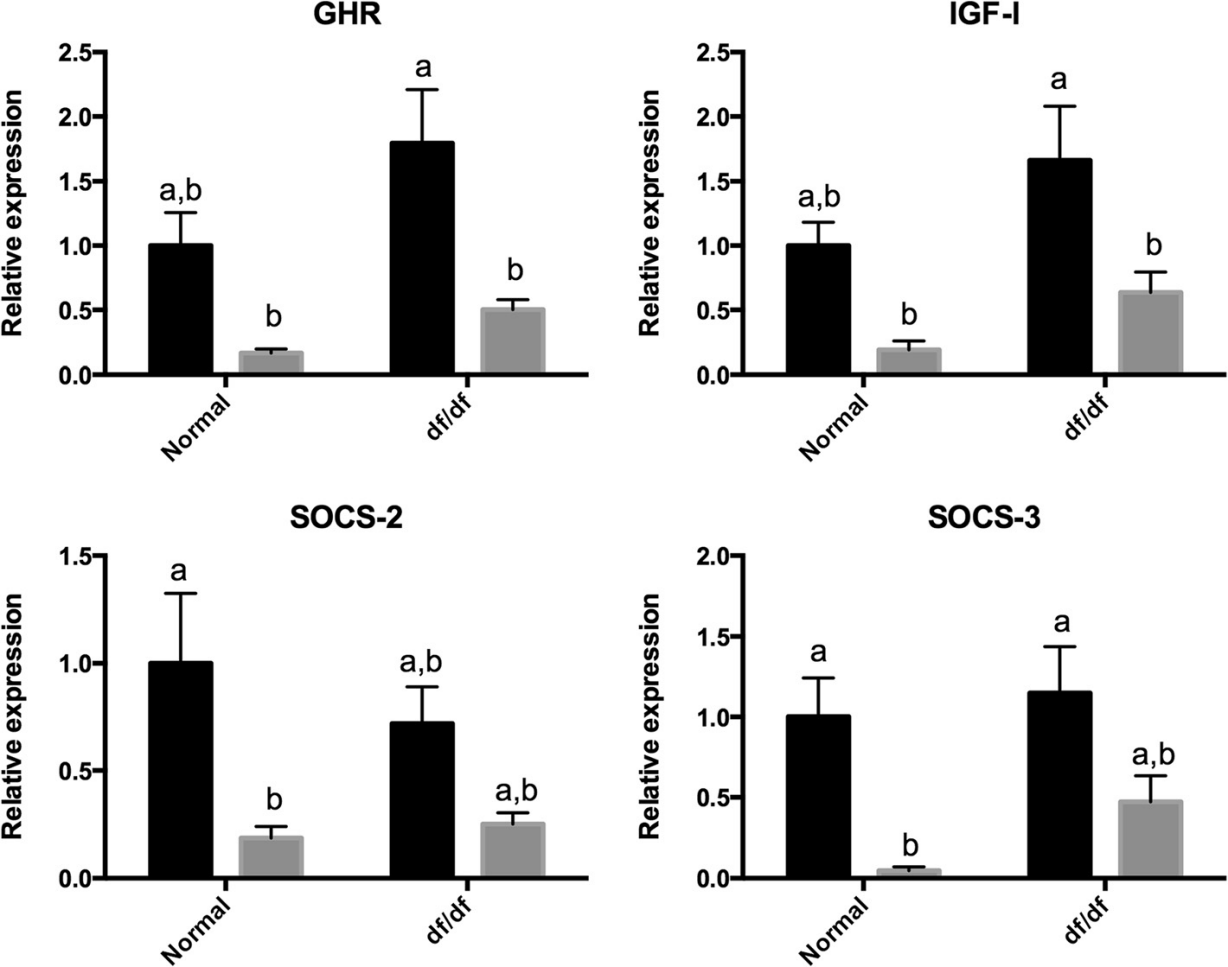
**Ovarian gene expression for genes in the GH signaling pathway in young (6 months–black bar) and old (22 months–grey bar) normal (N) and Ames dwarf (df/df) mice.** Different letter indicate significant differences (P<0.05). Expression of all genes showed was lower in old than younger mice (P<0.05).

**Figure 3:**
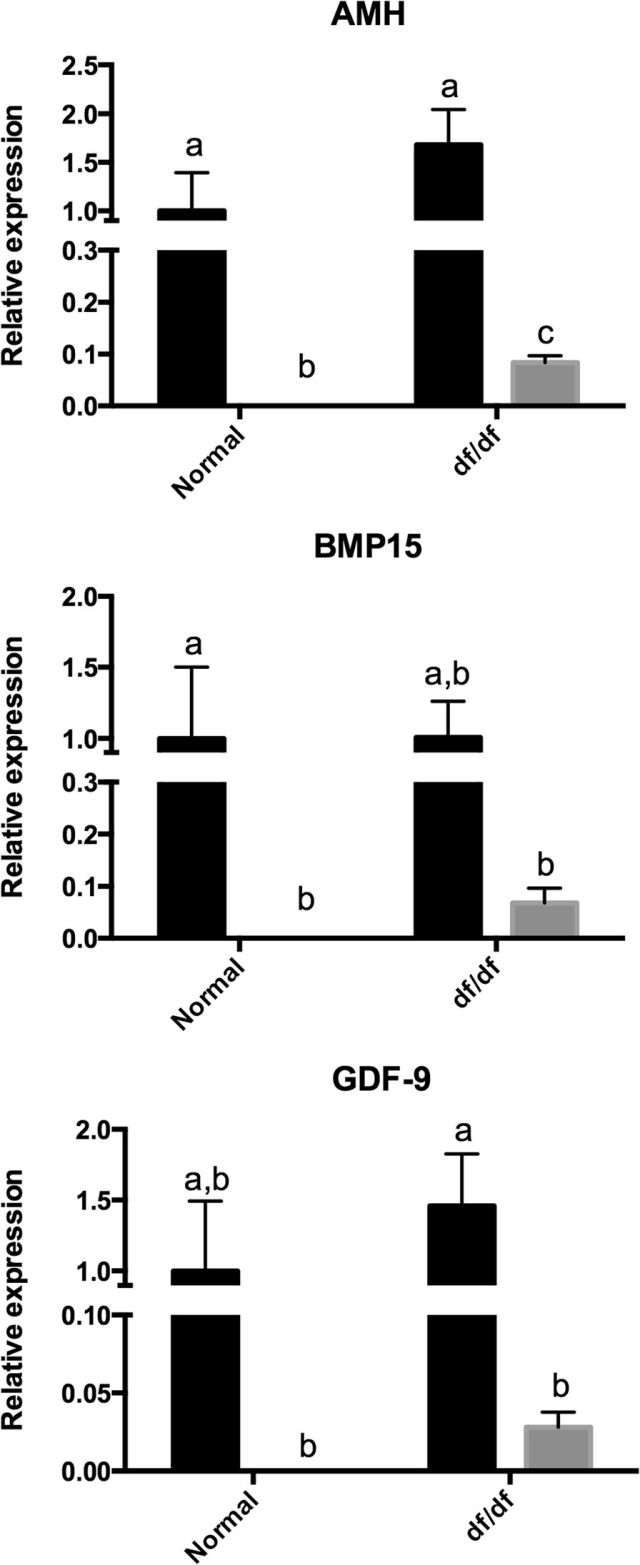
**Ovarian gene expression for genes indicating ovarian activity (AMH, GDF9 and BMP15) in young (6 months–black bar) and old (22 months–grey bar) normal (N) and Ames dwarf (df/df) mice.** Different letter indicate significant differences (P<0.05). Expression of all genes showed was lower in old than younger mice (P<0.05).

The expression of insulin receptor substrate 1 (*Irs1*), *Pi3k*, *Akt1*, *Mtor*, suppressor of cytokine signaling −2 (*Socs2*), *Socs3* was lower (P < 0.05) in older than younger N mice (Figures 1 and 2). Despite that, the expression of *Irs1*, *Pi3k*, *Akt1*, *Mtor*, *Socs2*, *Socs3* was not different (P > 0.05) between young and old df/df mice (Figures 1 and 2). The expression of *Foxo3a* was also lower (P < 0.05) in old than younger N and df/df mice. In addition, *Foxo3a* expression was higher (P < 0.05) in old df/df than N mice. The expression of *Ghr* and *Igf* was lower (P < 0.05) in old than young df/df mice. Despite that, expression *Ghr* and *Igf* was not different (P > 0.05) between young and old N mice. Expression of *Amh* was lower (P < 0.05) in old than young N and df/df mice. In addition Amh expression was higher (P = 0.0009) in df/df than N mice. Expression of *Bmp15* was lower (P < 0.05) in old than young N mice, but not different (P > 0.05) between old and young df/df mice. Expression of *Gdf9* was lower (P < 0.05) in old than young df/df mice, but not different (P > 0.05) between old and young N mice (Figures 3).

**Figure 4:**
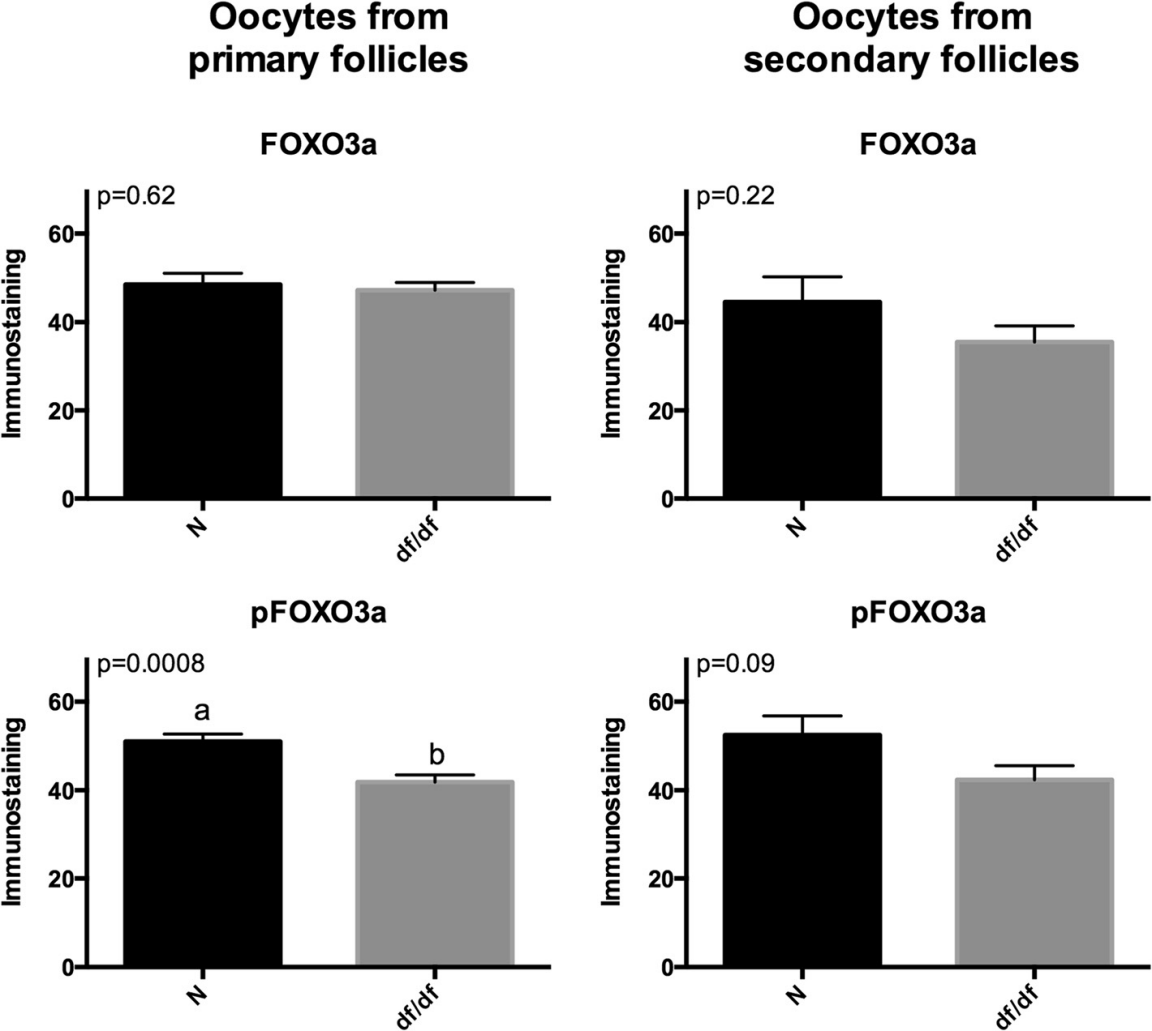
**Immunostaining for FOXO3 and p-FOXO3 in oocytes from primordial/primary and secondary follicles from 12-month old normal (N) and Ames dwarf (df/df) mice.**

**Figure 5:**
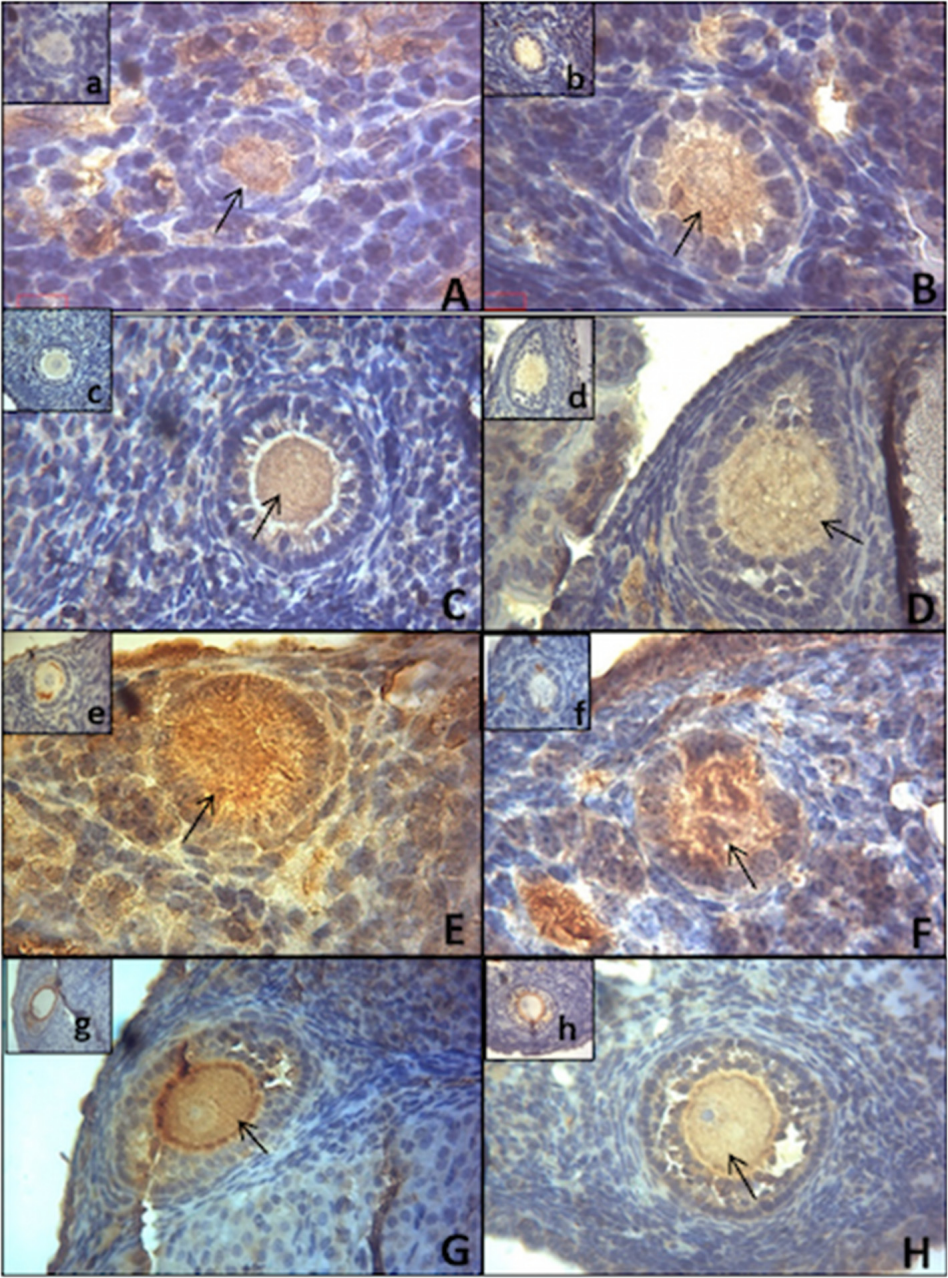
**Immunostaining (arrows) for FOXO3 (A-D) and p-FOXO3 (E-H) in oocytes included in primordial/primary follicles (OIPF) and oocytes included in secondary follicles (OISF) in N mice (A, C, E and G) and df/df mice (B, D, F and H).** Negative controls are represented in small Figures a-h. OIPF immunostained with FOXO3 in N and df/df mice, respectively. (Photomicrograph 100 X). **(C** and **D)** OISF immunostained with FOXO3 in N and df/df mice, respectively (photomicrograph 10 X). **(E** and **F)** OIPF immunostained with pFOXO3 in N and df/df mice, respectively. (Photomicrograph 100 X). **(G** and **H)** OISF immunostained with pFOXO3 in N and df/df mice, respectively. (Photomicrograph 10 X).

Foxo3 and p-Foxo3 immunostaining for OIPF and OISF in df/df and N mice is shown in Figures 4 and 5. It was observed that there was no difference between df/df and N mice for Foxo3 immunostaining in OIPF and OISF (P > 0.05). However, immunostaining for p-Foxo3 was higher in N vs df/df mice in OIPF (P = 0.0008), and there was no difference (P = 0.09) when comparing N vs Ames dwarf mice in OISF.

## Discussion

Expression of mRNA for *Irs1*, *Socs2*, *Socs3*, *Akt1*, *Pi3k*, *Mtor* and *Foxo3a* was lower in the ovaries of older mice of both genotypes. As mentioned earlier, activation of this pathway by growth factors is involved in the activation of primordial follicle growth by ultimately promoting phosphorylation and nuclear export of FOXO3a [20,22]. The lower expression of these genes in older mice points to this pathway as associated with the process of ovarian aging and follicle depletion. In fact, a recent study indicate that down-regulation of oocyte FOXO3a expression is essential for follicle progression from the primordial to later stages of development [34]. Older mice also had lower mRNA expression for *Amh*, *Gdf9* and *Bmp15*. The AMH is a member of the transforming growth factor β family (TGFβ) exclusively produced by granulosa cells (GC) of developing preantral and small antral follicles [35]. Serum AMH levels are widely used to estimate the size of the ovarian reserve [36]. GDF9 and BMP15 are also members of the TGFβ family and produced exclusively by growing oocytes in the mice ovary [37,38]. In the GDF9 null mice folliculogenesis does not progress beyond the primary stage [39], while BMP15 null mice had impaired oocyte maturation leading to infertility [40]. Therefore, both GDF9 and BMP15, in addition to AMH, are indicating lower ovarian activity in 22-month-old in relation to 6-month-old mice of both genotypes as we would expect during aging of the ovaries. Together, these results indicate that reduced ovarian activity with increasing age is associated with reduced expression of the members of the IRS1-Pi3k-Akt1-FOXO3 pathway.

Interestingly, expression of *Foxo3a* mRNA was 10-fold higher in the ovaries of old df/df mice when comparing to old N mice. FOXO3a is crucial in the maintenance of follicles in the primordial quiescent stage while not phosphorylated [22]. Therefore, the higher expression of *Foxo3a* indicates that follicles could be trapped in the primordial stage. This was confirmed by the observation that oocytes from primordial/primary follicles from Ames dwarf mice had a lower level of p-FOXO3 compared to N mice, although no difference in the level of FOXO3 was detected, indicating a lower level of activation of primordial follicles. It is known that phosphorylation of FOXO3a by the IRS-Pi3k-Akt pathway is what indicates its activation, therefore higher gene expression from members of the IRS-Pi3k-Akt pathway does not necessarily means more FOXO3a activation, because it depends on the level of activation of this pathway by insulin/IGF. Since Ames dwarf mice have strongly reduced insulin/IGF signaling [3], it is possible that this is the cause of the reduced level of p-FOXO3 in oocytes from Ames dwarf mice. This hypothesis agree with histological observations in long-living GH-resistant GHRKO mice and mice subjected to calorie restriction where a higher number of follicles in the primordial stage is observed concomitantly with less follicles in the primary, secondary and tertiary stages [16,18]. A recent paper has demonstrated that oocyte expression of a constitutively active form of FOXO3 in a transgenic mouse model is associated with a younger ovarian phenotype [41]. In this way FOXO3a is been referred as a potential determinant of the onset of menopause, as well as a potential agonist intervention to reduce the physiological decline in female fertility at later ages [41].

Therefore our study, in agreement with previous observations [41], indicates that higher expression of *Foxo3a* mRNA and decreased level of p-FOXO3 in oocytes from primordial/primary follicles, together with other genes in the pathway of insulin signaling as observed in df/df mice, is associated to increased expression of genes related to follicle recruitment and growth (*Amh*, *Gdf9* and *Bmp15*). *Amh* mRNA expression was about 80 times higher in old df/df when compared to old N mice. This result indicates that although normal old mice had severely depleted the ovarian follicular reserve (*Amh* mRNA expression about 900 times lower than young N mice), df/df mice still have some follicular activity (*Amh* mRNA expression about 20 times lower than young df/df mice). More studies are necessary to identify if the same pattern will be observed in GHRKO and calorie restricted N mice, but already points that modulation of the IRS-Pi3k-Akt pathway can modulate ovarian FOXO3a expression and therefore help in preventing follicle loss in older females.

Even though the liver is the main source of circulating IGF-I, many local tissues are able to produce IGF-I [42]. The importance of the endocrine versus the paracrine/autocrine ovarian IGF-I has been debated since the demonstration that GHRKO mice [15] and mice with liver-specific knockout of IGF-I [43] are able to reproduce, while the IGF-I null mice are infertile and fail to ovulate [44]. In this way, the data of the present study further support these findings by showing that df/df mice, that have no detectable levels of circulating GH and severely reduced IGF-I, are able to produce ovarian *Igf1* mRNA. The results indicate that ovarian *Igf1* mRNA expression is largely independent of GH as demonstrated in the df/df mice hippocampus [10] and in the ovaries of GHRKO mice [11].

## Conclusions

In sum, the present study indicates that lower expression of *Irs1*, *Socs2*, *Socs3*, *Akt1*, *Pi3k*, *Mtor* and *Foxo3a* mRNA in the ovaries of older mice of both genotypes is associated to a reduced ovarian activity revealed by lower expression of *Amh* mRNA. At the same time, ovaries of old df/df mice maintained higher expression of *Foxo3a* mRNA, which was associated to higher ovarian activity. We have shown that df/df females have a lower level of p-FOXO3 in oocytes from primordial/primary follicles, an important activator of follicular growth. Therefore, our study strongly indicates that Prop1^df^ mutation causes delayed ovarian aging.
